# Nephrotoxicity of Immune Checkpoint Inhibitors in Single and Combination Therapy—A Systematic and Critical Review

**DOI:** 10.3390/biomedicines13030711

**Published:** 2025-03-13

**Authors:** Javier Tascón, Alfredo G. Casanova, Laura Vicente-Vicente, Francisco J. López-Hernández, Ana I. Morales

**Affiliations:** 1 Toxicology Unit, Universidad de Salamanca (USAL), 37007 Salamanca, Spain; javiertascon_96@usal.es (J.T.); alfredogcp@usal.es (A.G.C.); lauravicente@usal.es (L.V.-V.); 2 Instituto de Investigación Biomédica de Salamanca (IBSAL), 37007 Salamanca, Spain; flopezher@usal.es; 3 Department of Physiology and Pharmacology, Universidad de Salamanca (USAL), 37007 Salamanca, Spain; 4 Group of Translational Research on Renal and Cardiovascular Diseases (TRECARD), 37007 Salamanca, Spain; 5 Group of Biomedical Research on Critical Care (BioCritic), 47005 Valladolid, Spain

**Keywords:** immune checkpoint inhibitors (ICIs), acute kidney injury, tubulointerstitial nephritis, nephrotoxicity, systematic review, cancer

## Abstract

**Background/Objectives:** Immune checkpoint inhibitors (ICIs) have generated a revolutionary approach in the treatment of cancer, but their effectiveness has been compromised by immune-related adverse events, including renal damage. Although rare, these effects are relevant because they have been related to poor patient prognoses. The objective of this review was to estimate the current incidence of nephrotoxicity in patients treated with single and double ICI therapies. **Methods:** A total of 1283 potential articles were identified, which were reduced to 50 after applying the exclusion and inclusion criteria. **Results:** This study reveals the increase in acute kidney injury associated with these drugs in the last decade and shows that, interestingly, combined therapies with ICIs does not lead to an increase in kidney damage compared with anti-CTLA-4. It also suggests that kidney damage could be underdiagnosed when it comes to interstitial nephritis, because definitive evidence requires a renal biopsy. **Conclusions:** In perspective, these conclusions could guide clinicians in making decisions for therapy personalization and highlight the need to search for new diagnostic systems that are more sensitive and specific to the type of damage and could replace the biopsy.

## 1. Introduction

Immune checkpoint inhibitors (ICIs) are monoclonal antibodies specifically directed at blocking immune checkpoints (molecules that regulate the balance between the elimination of foreign antigens and the recognition of autoantigens). These are IgG1-, IgG2-, and IgG4-type human or humanized immunoglobulins. Their pharmacological activity lies in blocking the inhibitory receptors found on T cells, thus allowing their activation and increasing the immune response. Consequently, ICIs reestablish the action of T cells against tumors [1,2].

ICIs have changed the treatment paradigm for several types of tumors with poor prognoses in the last decade. There are three ICI families available for clinical use. On the one hand are those that block immune checkpoints or inhibitory T-cell receptors, i.e., antibodies binding to and blocking the cytotoxic T lymphocyte-associated protein 4 (CTLA-4), representing the anti-CTLA-4 family, and those that prevent the binding of activating ligands to the programmed cell death protein 1 (PD-1), constituting the anti-PD-1 family. On the other hand, there is another family of antibodies that bind to and block the ligands found on tumor cells (PD-L1), which bind and activate PD-1 receptors in lymphocytes. These form the anti-PD-L1 antibody family [1,2].

In 2011, the United States Food and Drug Administration (FDA) approved the first ICI with a therapeutic indication to treat metastatic melanoma cancer. This anti-CTLA-4 antibody, named ipilimumab, was the pioneer preceding eight currently authorized antibodies: tremelimumab (anti-CTLA-4); nivolumab, pembrolizumab, cemiplimab, and dostarlimab (anti-PD-1); and avelumab, durvalumab, and atezolizumab (anti-PD-L1) [3,4].

The clinical use of these nine FDA-approved ICIs has expanded over more than nineteen different therapeutic indications [5]. Furthermore, combined ICI therapy poses a breakthrough in the treatment of some types of cancer, such as non-small-cell lung cancer and metastatic melanoma, in which the concomitant administration of ipilimumab (anti-CTLA-4) and nivolumab (anti-PD-1) increases the response rate and survival [6].

However, despite their innovative nature and the improvement in efficacy and safety, the use of these drugs has also unveiled a number of adverse events related to the immune system resulting from the boost in the immune response [7]. The incidence of adverse effects appears to be higher in patients receiving combined therapy compared with those receiving monotherapies, which would limit their use [8].

Kidney injury is one of the complications observed. Although not the commonest, renal toxicity is a relevant complication associated with a poorer prognosis [5]. Acute kidney injury (AKI), proteinuria, and electrolyte disorders are the main pathologies of the renal system associated with ICI treatment [9], and the most common pathological lesion that leads to AKI is tubulointerstitial nephritis (90% of cases) [10]. There are also other lesions associated with the autoreactivity of the immune system, such as glomerulonephritis [11].

Since the introduction of these drugs in 2011, the incidence of undesired renal effects has grown due to the increasing use and abundance of authorized ICIs and improved diagnosis [12]. Systematic reviews and meta-analyses dealing with the incidence of renal damage in patients treated with immune checkpoint inhibitors have been previously published, such as those by Liu et al. 2023 [13] and Xie et al. 2023 [14]. However, due to the limited experience accumulated since the recent introduction of these drugs, updated knowledge on incidence, based on the progressively increasing data available, may help guide decision making and optimize treatment. On these grounds, we aimed to estimate the current incidence of nephrotoxicity in cancer patients treated with single and double ICI therapies.

## 2. Materials and Methods

### 2.1. Retrieval of Published Studies

A bibliographic search of clinical studies published in Medline and the Web of Science databases until 4 September 2024 was carried out by entering the following keyword combinations: “(nephrotoxicity OR renal toxicity OR renal damage OR renal injury OR kidney injury OR kidney damage) AND (immune checkpoint inhibitor OR ICI OR ICPI OR ipilimumab OR tremelimumab OR nivolumab OR pembrolizumab OR cemiplimab OR atezolizumab OR durvalumab OR avelumab OR PD-1 OR CTLA-4 OR PD-L1)”. The filters used were: “Humans”, “English”.

### 2.2. Exclusion and Inclusion Criteria

Study selection was carried out following the PRISMA guidelines. Two researchers (J.T. and L.V.-V.) independently withdrew those articles that met any of the following exclusion criteria: (1) preclinical studies, (2) reviews, protocols, communications, and letters to the editor, (3) written in a language other than English, (4) full text not available, and (5) studies evaluating the renal safety of combined therapies of ICIs with other antineoplastic drugs. After that, they selected those studies that met all of the following inclusion criteria: (1) clinical studies evaluating at least one ICI drug and (2) evaluating at least one kidney damage parameter: AKI or nephritis (independently for each ICI family). Discrepancies in article selection were resolved by a third researcher (A.G.C.). The screening was carried out with the online tool “Systematic review facility” [15].

This protocol was registered with the International Platform of Registered Systematic Review and Meta-Analysis Protocols (INPLASY registration number: INPLASY202510053) [16].

### 2.3. Data Extraction

The following data were extracted from each included work: first author’s name, publication year, study design and duration, geographic location, number of patients included, patient characteristics, tumor type and stage, ICI drugs, posology, route of administration, duration of treatment, Jadad or MINORS scale, and number of patients who presented alterations in any of the following parameters of kidney damage: AKI (elevation of plasma creatinine or renal failure) and nephritis. Study quality was assessed with the Jadad scale [17] (for prospective randomized studies) or the MINORS scale [18] (for retrospective or prospective, non-randomized studies). A threshold score of 2 out of 5 (in the Jadad scale) or 7 out of 16 (in the MINORS scale) was established for inclusion in this review.

### 2.4. Calculation of Incidence

In each clinical study, percentage incidence of AKI and nephritis were calculated according to the following formula:

$$Incidence\;\left(\%\right)=\frac{Number\;of\;cases\;(AKI\;or\;nephritis)}{Total\;number\;of\;patients}\times 100$$

The weighted mean and the standard error of the mean of AKI and nephritis incidence in ICI families and in the anti-PD-1 + anti-CTLA-4 combination were also calculated. In addition, the weighted mean and the standard error of the mean of the incidence of the kidney damage parameters of nivolumab (anti-PD-1), pembrolizumab (anti-PD-1), atezolizumab (anti-PD-L1), durvalumab (anti-PD-L1), ipilimumab (anti-CTLA-4), tremelimumab (anti-CTLA-4), nivolumab + ipilimumab (anti-PD-1 + anti-CTLA-4), and pembrolizumab + ipilimumab (anti-PD-1 + anti-CTLA-4) were also calculated. Figures design was carried out with Microsoft Office 365^®^ software (Redmond, WA, USA).

## 3. Results

**Figure 1:**
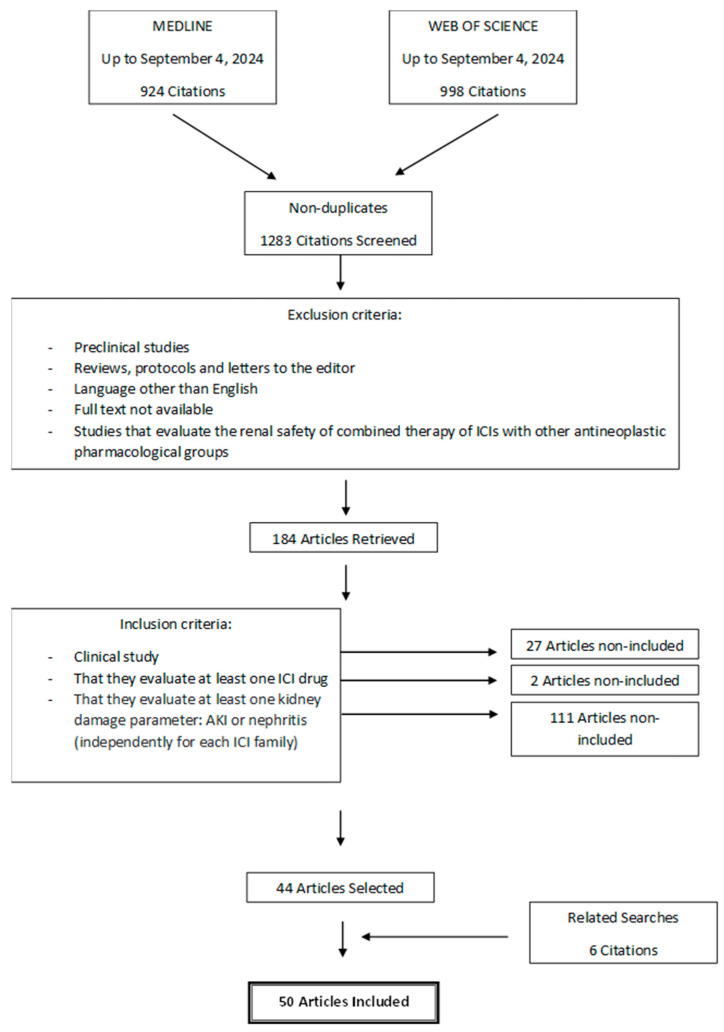
Flow chart of search and selection of studies carried out in accordance with the PRISMA guidelines.

The flow chart describing the study search process and definitive inclusion of cited references is presented in Figure 1. A total of 1283 potential articles were identified, which, after applying the exclusion and inclusion criteria, was reduced to 44 selected articles. During the data extraction stage of the studies, 6 new articles were included from related searches, adding up to a total of 50 clinical studies. The descriptive data of the 50 clinical studies finally included in the review are shown in Table 1.

The 50 clinical studies analyzed in this review were mostly retrospective studies and a few prospective and randomized clinical trials carried out in different hospitals worldwide, mostly with metastatic melanoma and metastatic renal cancer patients, but also with patients with other types of cancer such as non-small-cell lung cancer. The most commonly used ICIs were nivolumab and pembrolizumab (anti-PD-1), ipilimumab (anti-CTLA-4), and the combined therapy of nivolumab + ipilimumab. These drugs were administered intravenously for several cycles, essentially every 2 or 3 weeks. The most frequently quantified parameter to evaluate renal function was the elevation of plasma creatinine.

The incidence of AKI was practically similar in the groups of anti-PD-1 (5.32%) and anti-PD-L1 (5.25%) but increased in patients treated with anti-CTLA-4 drugs (7.83%). Yet, it is noteworthy that ipilimumab had a higher incidence of AKI than tremelimumab (7.87% versus 4.35%, respectively). The higher incidence of AKI observed with ipilimumab may be related to the longer period of time that this drug has been used in clinical practice and the greater number of observational studies available (ipilimumab was the first ICI approved by the FDA in 2011, in contrast to tremelimumab, which was approved in 2022) [4]. On the other hand, the incidence of AKI in the combined therapy group (anti-PD-1 + anti-CTLA-4) was 5.58% (Table 2).

The mean incidence of nephritis in patients treated with anti-PD-1 did not exceed 1.5% (Table 2). However, the clinical study carried out by O’Reilly et al. in 2019 reported an incidence of nephritis of 3.39% in patients treated with ipilimumab [45]. In relation to the combined drug therapy (anti-PD-1 + anti-CTLA-4), the mean incidence of nephritis reached values around 2%, although the study carried out by Tykody et al. in 2022 evidenced an incidence of nephritis in patients treated with nivolumab + ipilimumab of 3.85% [62].

## 4. Discussion

ICIs have revolutionized the treatment of certain types of tumors, but their effectiveness has been limited by adverse effects related to the immune system, including renal effects [9,68]. Furthermore, numerous clinical studies have validated their advantageous efficacy profile not only when administered as monotherapy or in combination, but also when combined with chemotherapy, which has led to an increase in the survival of cancer patients [69].

Our study reveals that the incidence of AKI has kept growing in recent years (anti-PD-1 ~5%, anti-PD-L1 ~5%, anti-CTLA-4 ~8%, anti-PD-1 + anti-CTLA-4 ~6%), leaving outdated the previous incidence values of 2–3% for monotherapies and 5% for combined therapies [70]. The actual incidence of nephrotoxicity might be even higher, and damage is likely underdiagnosed in subclinical, asymptomatic, and mild cases due to the poor and sub-optimally sensitive diagnosis technology. In fact, current diagnosis of AKI is curtailed by the known limitations in sensitivity and specificity of the standard biomarker, plasma creatinine [71].

According to the existing literature, nephritis is the cause of 90% of ICI nephrotoxicity cases [10]. However, in this study we did not find incidences of nephritis that were too high (between 1 and 3%) for what would be expected taking into account that nephritis is the main cause of nephrotoxicity. This suggests that nephritis could be underestimated in many studies because definitive diagnosis is made by biopsy [70], which is medically restricted because of its invasive nature and potential complications [72].

ICIs’ toxicity is related to their pharmacological action, namely the release of the physiological blockages that regulate the immune response [9,68]. However, the anti-CTLA-4 and anti-PD-1/anti-PD-L1 families act differently because they differ in the location of their molecular targets, the stage of T-cell activation they facilitate, and the signaling pathways involved [73]. This could be the reason why the anti-PD-1 family is more effective and less toxic than the anti-CTLA-4 family, a fact that has been observed in various types of cancer, such as advanced melanoma [74,75,76]. This systematic review agrees with previous studies that show a higher incidence of adverse effects (also AKI) in patients treated with anti-CTLA-4 drugs compared with those treated with anti-PD-1.

The joint administration of anti-PD-1/anti-PD-L1 and anti-CTLA-4 could provide synergistic efficacy due to the combination of their different mechanisms of action, which could justify a higher response rate and survival of patients. However, this increase in efficacy could also entail an upsurge in adverse effects [6] that limit their use. With regard to nephrotoxicity, our study contrasts with the existing literature [6,77]. The incidence of AKI following the single anti-CTLA-4 treatment is 7.83%, while after combined therapy of anti-PD-1 + anti-CTLA-4, it is not greater (5.58%). These data suggest that the combined therapy would provide a benefit from an improved antitumor efficacy with no additional nephrotoxic burden.

The findings highlighted in this review updating the kidney damage incidence data reveal the favorable benefit–risk balance of combined ICI therapies in order to improve clinical decision making [23,24,35,63]. In many cases, this could be a successful therapeutic strategy, which would allow two families of ICIs to be included in the therapy without increasing the risk of suffering adverse renal effects (the incidence of kidney damage is not greater than after a single anti-CTLA-4). This is an important piece of information for clinical decision making, since recent systematic reviews, such as that by Xie et al., 2023 [14], report higher incidences of renal damage for combined ICI therapy than for monotherapies.

The different toxicities of these drug families could be related to the mechanism of action of each of them. The blockade carried out by anti-CTLA-4 on the CTLA-4 receptor at the lymph node level alters the quiescence phase of T cells and promotes their activation. This blockade of the inhibitory receptor restores the activating signal of the CD28 costimulatory receptor. Consequently, T cells are activated, and the adaptive immune response against tumor cells is regenerated [9,78]. However, PD-1/PD-L1 blockade occurs at a different site than CTLA-4 blockade. It affects the effector phase of the adaptive immune response and takes place in the tumor microenvironment [68]. In this sense, the T cell–CD28 costimulatory receptor signaling pathway is reestablished. As a result, the production of cytokines, the proliferation of T lymphocytes, and the cytotoxic activity of the T cell is promoted [9,73,78]. This would translate, in addition to the therapeutic response, into the appearance of adverse effects, including kidney effects, due to the attack of autoantigens as a consequence of the overactivation of the lymphocyte system.

**Figure 2:**
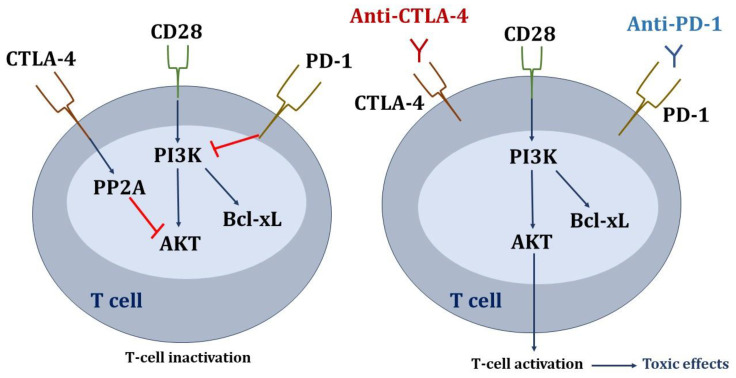
Signaling pathways affected in T lymphocytes due to the inhibition of check points. Blue arrows mean activation, and red lines mean inhibition.

Regarding the signaling pathways affected in T lymphocytes (Figure 2), the inhibitory signals through PD-1 and CTLA-4 converge on serine-threonine kinase (AKT), although the inhibition pathways are different. Parry RV et al. demonstrated that PD-1 signaling blocks CD28-mediated activation of inositolphosphatidyl-3-kinase (PI3K) and AKT. Inhibitory CTLA-4 signaling preserves PI3K activity but inhibits AKT directly through the activation of the phosphatase protein PP2A. Therefore, anti-CTLA-4 and anti-PD-1 antibodies would act at different levels in the T-cell signaling cascade in order to stimulate immune activation [78].

Recently, anti-CTLA-4 drugs have been called “immune enhancers”, and anti-PD-1/anti-PD-L1 drugs “immune normalizers” [79]. In this sense, anti-PD-1/anti-PD-L1 would cause a normalization of immunity, while anti-CTLA-4 would enhance it. This fact could justify the greater renal toxicity produced by anti-CTLA-4 drugs with respect to anti-PD-1/anti-PD-L1.

**Figure 3:**
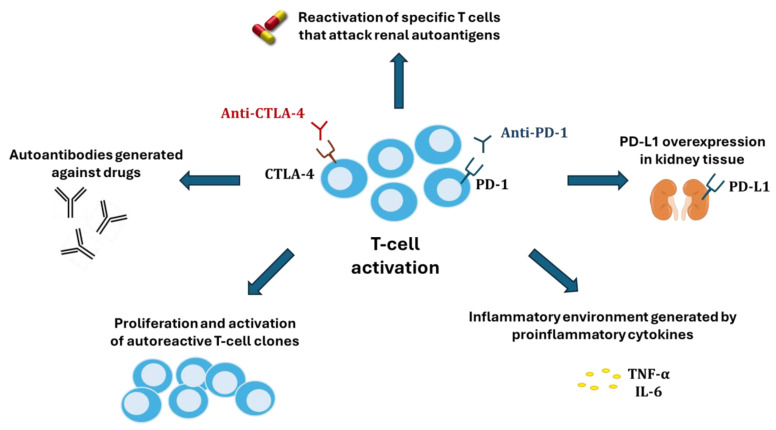
Mechanisms of kidney damage associated with immune checkpoint inhibitors.

Several hypotheses have been proposed to explain the nephrotoxicity of ICIs (Figure 3). One of these is the reactivation of specific T cells (in the latency period) that had been generated after the administration of other nephrotoxic drugs (proton pump inhibitors, non-steroidal anti-inflammatory drugs, and antibiotics) [80] and which could now attack renal autoantigens. Alternatively, ICIs could cause an abnormal proliferation and activation of autoreactive T-cell clones or the production of autoantibodies by B cells recognizing self-antigens in tubular epithelial cells, mesangial cells, or podocytes, until then tolerated by the immune system [9]. A third potential mechanism would be the inflammatory environment generated within kidney tissue by proinflammatory cytokines secreted by T cells. Finally, because renal tubular cells constitutively express PD-L1 to avoid T cell-mediated autoimmunity, blocking its interaction with PD-1 in T cells could disable the defense and facilitate cytotoxic injury [81].

Our study has encountered a few caveats that limit coherent comparison among the studies, which need to be addressed in future studies reporting ICI nephrotoxicity. For instance, the absence of a standard definition and diagnostic criteria for ICI nephropathy prevents the full leverage and integration of data from multiple studies. An international consensus initiative is necessary in this regard.

In conclusion, this review reveals an increased incidence of AKI associated with ICI drugs that should alert clinicians, especially in patients at risk. On the other hand, it also shows that combined ICI therapy is not more nephrotoxic than single anti-CTLA-4 therapy, underpinning a better efficacy-to-safety relation than previously reported. Finally, it suggests that kidney damage could be underdiagnosed because of the absence of reliable, non-invasive technology for differential diagnosis, as conclusive diagnosis still requires a biopsy.

There is a need to search for new, earlier, and more sensitive diagnostic systems. In this sense, new, preferably urinary, biomarkers or, more probably, collections of biomarkers are sought to compose etiological fingerprints with which to differentiate underlying damage patterns through a liquid biopsy. In addition, longer term follow-up studies should be conducted to monitor the evolution and duration of renal side effects and the sequelae of single and double therapies with traditional and new diagnostic methods under a unified definition of ICI nephropathy that allows for cross-study data standardization and comparison.

## Figures and Tables

**Table 1:** Descriptive characteristics of the studies included in the systematic review. Acute kidney injury (AKI).

Study Identification	Design	Location	Duration of Recruitment	Tumor Type and Stage	Number of Patients Initially Included	Patient Characteristics	Drug	Posology	Parameters of Renal Injury		Jadad/MINORS Scale
									**AKI**	**Nephritis**	
Abdelrahim et al., 2021 [19]	Retrospective study	USA	1 January 2010 to 12 November 2019	Metastatic or advanced melanoma	1664	Black, Hispanic, Asian, and White ethnicity. Median age 63 years.	Nivolumab, pembrolizumab, atezolizumab, ipilimumab, nivolumab + ipilimumab, pembrolizumab + ipilimumab	Not specified	Yes	No	10 of 16 (MINORS)
Antonia et al., 2016 [20]	Multicenter, open-label, two-stage, multi-arm phase 1/2 trial	Finland, Germany, Italy, Spain, UK, and USA	18 November 2013 to 28 July 2015	Extensive stage non-small-cell lung cancer	216	White, Black or African American, and other ethnicities. Median age 63 years.	Nivolumab, nivolumab + ipilimumab	3 mg/kg every 2 weeks 1 mg/kg + 1 mg/kg. Depending on tolerability, 1 mg/kg + 3 mg/kg or 3 mg/kg + 1 mg/kg every 3 weeks	Yes	No	12 of 16 (MINORS)
Apolo et al., 2020 [21]	Phase 1, open-label, multicohort trial	USA	3 September 2014 to 15 March 2016	Advanced or metastatic urothelial cancer	249	Ethnicity not specified. Median age 69 years.	Avelumab	10 mg/kg every 2 weeks	Yes	No	10 of 16 (MINORS)
Atkins et al., 2018 [22]	Open-label, multicohort, phase 1b study	USA	2 April 2014 to 18 November 2014	Advanced melanoma and advanced renal cell carcinoma	39	Ethnicity not specified. Median age 60.5 years.	Pembrolizumab + ipilimumab	2 mg/kg + 1 mg/kg every 3 weeks	Yes	No	3 of 5 (Jadad)
Atkins et al. 2023 [23]	Single-arm, open-label, non-randomized, multicenter, phase 2 study	USA	May 2017 to December 2019	Metastatic renal cancer	35	Ethnicity not specified. Median age 65 years.	Nivolumab nivolumab + ipilimumab	240 mg every 2 weeks 3 mg/kg and 1 mg/kg i.v. every 12 weeks	Yes	No	11 of 16 (MINORS)
Blas et al. 2024 [24]	Retrospective study	USA	2013 to 2022	Metastatic renal cancer	2921	Ethnicity not specified. Median age 65 years.	Nivolumab + ipilimumab	Not specified	Yes	Yes	10 of 16 (MINORS)
Campbell et al., 2021 [25]	Pilot study	USA	7 November 2016 to 25 October 2018	Metastatic renal cancer	30	Hispanic or Latino and White or Caucasian ethnicity. Median age 64 years.	Tremelimumab	10 mg/kg every 4 weeks	Yes	No	2 of 5 (Jadad)
Cortazar et al., 2020 [11]	Multicenter, retrospective cohort study	USA and Canada	Not specified	Melanoma, lung, genitourinary, others	414	White, Black, and Asian ethnicity. Median age 67 years.	Nivolumab, pembrolizumab, ipilimumab	Not specified	Yes	No	9 of 16 (MINORS)
Dizman et al., 2022 [26]	Single-center, open-label, investigator-initiated trial	USA	22 April 2019 to 30 December 2020	Metastatic renal cancer	30	White and Asian ethnicity. Median age 64 years.	Nivolumab + ipilimumab	3 mg/kg + 1 mg/kg every 3 weeks followed by nivolumab monotherapy at 480 mg monthly	Yes	No	3 of 5 (Jadad)
Espi et al., 2021 [27]	Retrospective analysis	France	January 2015 to July 2017	Advanced melanoma, non-small-cell lung cancer, and urologic cancers	352	Ethnicity not specified. Median age 67 years.	Nivolumab, pembrolizumab	Not specified	Yes	No	10 of 16 (MINORS)
Flippot et al., 2019 [28]	Phase 2 trial	France	12 February 2016 to 27 July 2017	Brain metastases from renal cell carcinoma	73	Ethnicity not specified. Median age 59.5 years.	Nivolumab	3 mg/kg every 2 weeks	Yes	No	11 of 16 (MINORS)
George et al., 2022 [29]	Largely community-based, multicohort, open-label, phase 3b/4 trial	USA	January 2017 to March 2018	Advanced renal cell carcinoma	106	White, Black or African American, and other ethnicities. Median age 64.5 years.	Nivolumab + ipilimumab	6 mg/kg + 1 mg/kg every 8 weeks	Yes	Yes	13 of 16 (MINORS)
Goldberg et al., 2020 [30]	Two-arm phase 2 trial	USA	31 March 2014 to 21 May 2018	Brain metastases from stage IV non-small cell lung cancer	42	Ethnicity not specified. Median age 60 years	Pembrolizumab	10 mg/kg every 2 weeks	Yes	No	10 of 16 (MINORS)
Grimm et al., 2022 [31]	Prospective, observational, multicenter study	Germany	October 2016 to December 2018	Advanced renal cell carcinoma	228	Ethnicity not specified. Median age 70 years	Nivolumab	3 mg/kg every 2 weeks, 240 mg every 2 weeks, or 480 mg every 4 weeks	Yes	No	11 of 16 (MINORS)
Gul et al., 2020 [32]	Retrospective study	USA	Not specified	Metastatic renal cell carcinoma	45	Ethnicity not specified. Median age 62 years	Nivolumab + ipilimumab	Not specified	No	Yes	10 of 16 (MINORS)
Hammers et al., 2017 [33]	Multicenter, open-label, phase 1 study	Not specified	February 2012 to May 2014	Metastatic renal cell carcinoma	194	White, Asian, Black or African American, and other ethnicities. Median age 55 years.	Nivolumab + ipilimumab	3 mg/kg + 1 mg/kg, 1 mg/kg + 3 mg/kg, or 3 mg/kg + 3 mg/kg every 3 weeks	Yes	No	2 of 5 (Jadad)
Hofmann et al., 2016 [34]	Retrospective study	Germany and Switzerland	Not specified	Metastatic melanoma	496	Ethnicity not specified. Median age 59 years.	Nivolumab, pembrolizumab	3 mg/kg every 2 weeks 2 mg/kg every 3 weeks	No	Yes	9 of 16 (MINORS)
Izumi et al. 2023 [35]	Retrospective study	Japan	September 2018 to February 2021	Metastatic renal cell carcinoma	131	Ethnicity not specified. Median age 65 years.	Nivolumab + ipilimumab	240 mg and 1 mg/kg i.v every 3 weeks	Yes	No	10 of 16 (MINORS)
Julien et al., 2020 [36]	Phase 1/2, open-label study	USA, Spain, Hong Kong, and Singapore	Until March 2017	Advanced hepatocellular carcinoma	262	Ethnicity not specified. Median age 63 years.	Nivolumab	0.1 mg/kg, 0.3 mg/kg, 1.0 mg/kg, 3.0 mg/kg, or 10.0 mg/kg every 2 weeks	Yes	Yes	11 of 16 (MINORS)
Kanz et al., 2016 [37]	Multicenter, retrospective study	Guyana	6 January 2013 to 31 December 2015	Advanced renal cell carcinoma, melanoma, non-small-cell lung cancer, small-cell lung cancer, and urothelial bladder cancer	27	Ethnicity not specified. Median age 69 years.	Nivolumab, pembrolizumab	Not specified	No	Yes	9 of 16 (MINORS)
Martini et al., 2018 [38]	Retrospective cohort study	USA, Spain, Brazil	Not specified	Metastatic renal cell carcinoma	19	Ethnicity not specified. Median age 63 years.	Anti-PD-1 Anti-PD-L1	Not specified	No	Yes	7 of 16 (MINORS)
Massard et al., 2016 [39]	Phase 1/2 multicenter, open-label study	Not specified	28 August 2014 to 10 November 2015	Advanced urothelial bladder cancer	61	White, Asian, Black or African American, and other ethnicities. Median age 66 years.	Durvalumab	10 mg/kg every 2 weeks	Yes	No	11 of 16 (MINORS)
McFarlane et al., 2020 [40]	Open-label, phase 3b/4 study	USA	December 2015 to December 2016	Advanced clear-cell renal cell carcinoma	97	Ethnicity not specified. Median age 64.7 years.	Nivolumab	240 mg every 2 weeks	No	Yes	10 of 16 (MINORS)
Meraz-Muñoz et al., 2020 [41]	Retrospective cohort study	Canada	1 January 2010 to 1 January 2017	Metastatic melanoma, lung, genitourinary, lymphoma, ovarian, and colon cancer	309	Ethnicity not specified. Median age 63 years.	Nivolumab, pembrolizumab, ipilimumab, nivolumab + ipilimumab	Not specified	Yes	No	9 of 16 (MINORS)
Mok et al., 2019 [42]	Randomized, open-label, controlled, phase 3 trial	Argentina, Brazil, Bulgaria, Canada, Chile, China and Hong Kong Special Administrative Region, Colombia, Czech Republic, Estonia, Guatemala, Hungary, Japan, Latvia, Lithuania, Malaysia, Mexico, Peru, Philippines, Poland, Portugal, Romania, Russia, South Africa, South Korea, Sweden, Switzerland, Taiwan, Thailand, Turkey, Ukraine, and Vietnam	19 December 2014 to 6 March 2017	Locally advanced or metastatic non-small-cell lung cancer	1275	Ethnicity not specified. Median age 63 years.	Pembrolizumab	200 mg every 3 weeks	Yes	No	3 of 5 (Jadad)
Mourey et al. 2024 [43]	Phase 2 study	France	Not specified	Metastatic renal cancer	720	Ethnicity not specified. Median age 70 years.	Nivolumab	3 mg/kg every 2 weeks	Yes	No	8 of 16 (MINORS)
Noronha et al., 2021 [44]	Single-center retrospective analysis	India	August 2015 to November 2018	Advanced head and neck cancer, lung cancer, adenocarcinoma, squamous cell carcinoma, small-cell lung cancer, renal cell carcinoma, urothelial carcinoma, malignant mesothelioma (advanced non-melanoma solid tumors)	155	Ethnicity not specified. Median age 57 years.	Nivolumab, pembrolizumab	3 mg/kg every 2 weeks/240 mg flat dose every 2 weeks 200 mg flat dose every 3 weeks	No	Yes	9 of 16 (MINORS)
O’Reilly et al., 2020 [45]	Retrospective study	United Kingdom	May 2017 to August 2017	Metastatic melanoma	84	Ethnicity not specified. Median age 65 years.	Nivolumab, pembrolizumab, ipilimumab	Not specified	No	Yes	10 of 16 (MINORS)
Polkowska et al., 2019 [46]	Retrospective observational study	Poland	March 2013 to October 2016	Metastatic melanoma	1170	Ethnicity not specified. Median age 61 years.	Ipilimumab	Not specified	Yes	No	10 of 16 (MINORS)
Powles et al., 2020 [47]	Open-label, randomized, controlled, phase 3 trial	China	24 November 2015 to 21 March 2017	Locally advanced or metastatic urothelial carcinoma	1032	White or Caucasian, Asian, Black or African American, and other ethnicities. Median age 67.5 years.	Durvalumab, durvalumab + tremelimumab	1500 mg every 4 weeks 1500 mg + 75 mg every 4 weeks	Yes	No	3 of 5 (Jadad)
Powles et al., 2022 [48]	Multicenter, randomized, double blind, placebo-controlled, phase 3 trial	North America, South America, Europe, Asia, and Australia	30 June 2017 to 20 September 2019	Advanced clear-cell renal cell carcinoma	994	Hispanic or Latino and not Hispanic ethnicity. Median age 60 years.	Pembrolizumab	200 mg every 3 weeks	Yes	Yes	5 of 5 (Jadad)
Raghav et al., 2022 [49]	Phase 2 study, open-label, single-center, multicohort trial.	USA	29 August 2016 to 29 June 2020	Advanced solid tumors	29	Ethnicity not specified. Median age 59 years.	Pembrolizumab	200 mg every 3 weeks	Yes	No	10 of 16 (MINORS)
Rassy et al., 2022 [50]	Retrospective analysis	France	February 2016 to July 2017	Advanced renal cell carcinoma	729	Ethnicity not specified. Median age 64 years.	Nivolumab	3 mg/kg every 2 weeks	Yes	No	10 of 16 (MINORS)
Ready et al., 2023 [51]	Phase 3B, multicenter, open-label, single-arm, multicohort safety study	North America, Europe, and South America	November 2016 to 19 February 2021	Metastatic non-small- cell lung cancer	391	White and Black ethnicity. Median age 65 years.	Nivolumab + ipilimumab	240 mg every 2 weeks + 1 mg/kg every 6 weeks	No	Yes	12 of 16 (MINORS)
Reck et al., 2016 [52]	Open-label, phase 3 trial	Not specified	19 September 2014 to 29 October 2015	Advanced non-small-cell lung cancer	305	Ethnicity not specified. Median age 64.5 years.	Pembrolizumab	200 mg every 3 weeks	Yes	No	2 of 5 (Jadad)
Rini et al., 2011 [53]	Phase 1, open-label, multicenter, dose-escalation study	Not specified	December 2006 to January 2009	Metastatic renal cancer	28	Ethnicity not specified. Median age 60 years.	Tremelimumab	6 mg/kg, 10 mg/kg, or 15 mg/kg every 12 weeks	Yes	No	11 of 16 (MINORS)
Seethapathy et al., 2019 [54]	Retrospective observational cohort study	USA	May 2011 to December 2016	Not specified	1016	White, Black, Hispanic, Asian, and other ethnicities. Median age 65 years.	Nivolumab, pembrolizumab, atezolizumab, avelumab, durvalumab, ipilimumab, nivolumab + ipilimumab	Not specified	Yes	No	10 of 16 (MINORS)
Sharma et al., 2016 [55]	Multicenter, open-label, two-stage, multi-arm, phase 1/2 study	Finland, Germany, Spain, UK, and USA	June 2014 to April 2015	Metastatic urothelial carcinoma	86	White, Black or African American, Asian, and other ethnicities. Median age 65.5 years.	Nivolumab	3 mg/kg every 2 weeks	Yes	No	11 of 16 (MINORS)
Spillane et al., 2020 [56]	Retrospective observational study	USA	1 January 2011 to 31 August 2018	Metastatic melanoma	2407	Ethnicity not specified Median age 73.75 years.	Nivolumab, pembrolizumab, ipilimumab, nivolumab + ipilimumab	Not specified	Yes	No	10 of 16 (MINORS)
Sternberg et al., 2019 [57]	Single-arm multicenter international open-label phase 3B safety study	Europe, Asia, South America, Australia, and Canada	November 2016 to March 2018	Locally advanced or metastatic urothelial or nonurothelial carcinoma of the urinary tract	997	Ethnicity not specified. Median age 68 years.	Atezolizumab	1200 mg every 3 weeks	Yes	No	10 of 16 (MINORS)
Sukari et al., 2019 [58]	Retrospective study	USA	3 August 2011 to 31 August 2016	Advanced non-small-cell lung cancer, renal cell carcinoma, Hodgkin’s lymphomas, head and neck squamous cell carcinoma, small-cell lung cancer	168	Ethnicity not specified. Median age 63 years.	Nivolumab, pembrolizumab	Not specified	Yes	No	10 of 16 (MINORS)
Tachibana et al., 2021 [59]	Retrospective study	Japan	December 2015 to May 2020	Advanced papillary renal cell carcinoma	30	Ethnicity not specified. Median age 67 years.	Nivolumab + ipilimumab	Not specified	Yes	No	10 of 16 (MINORS)
Tio et al., 2018 [60]	Retrospective cohort study	Not specified	July 2014 to March 2017	Advanced melanoma, urothelial carcinoma, renal cell carcinoma, mesothelioma, hepatocellular carcinoma, non-small-cell lung cancer, gastric cancer, glioblastoma	46	Ethnicity not specified. Median age 60 years.	Nivolumab, pembrolizumab, atezolizumab, nivolumab + ipilimumab, pembrolizumab + ipilimumab	Not specified	Yes	Yes	9 of 16 (MINORS)
Tomita et al., 2019 [61]	Phase 3, randomized, open-label study	Japan	9 October 2012 to 14 March 2014	Advanced renal cell carcinoma	821	Ethnicity and median age not specified.	Nivolumab	3 mg/kg every 2 weeks	Yes	No	3 of 5 (Jadad)
Tykodi et al., 2022 [62]	Non-randomized, open-label, multicohort, phase 3b/4 clinical trial	USA	Not specified	Advanced non-clear-cell renal cell carcinoma	52	Ethnicity not specified. Median age 64 years.	Nivolumab + ipilimumab	3 mg/kg + 1 mg/kg every 3 weeks	No	Yes	9 of 16 (MINORS)
Vasudev et al. 2024 [63]	Phase 2, multicenter, randomized, controlled trial	United Kingdom	March 2018 to January 2020	Advanced renal cell carcinoma	192	Ethnicity and median age not specified.	Nivolumab + ipilimumab	3 mg/kg and 1 mg/kg i.v. every 12 weeks	Yes	No	3 of 5 (Jadad)
Verhaart et al., 2021 [64]	Retrospective analysis	The Netherlands	March 2016 to January 2018	Advanced renal cell carcinoma	264	Ethnicity not specified. Median age 65 years.	Nivolumab	3 mg/kg every 2 weeks	No	Yes	10 of 16 (MINORS)
Vogelzang et al., 2020 [65]	Open-label phase 3b/4 study	USA	December 2015 to December 2016	Advanced non-clear-cell renal cell carcinoma	44	Ethnicity not specified Median age 62 years.	Nivolumab	240 mg every 2 weeks	No	Yes	10 of 16 (MINORS)
Zhao et al., 2021 [66]	Retrospective study	Singapore	November 2016 to April 2020	Advanced renal cell carcinoma	32	Chinese, Indian, Malay, and Caucasian ethnicities. Median age 64 years.	Nivolumab	1.7 mg/kg and 2.7 mg/kg every 2 weeks	No	Yes	9 of 16 (MINORS)
Zhou et al. 2024 [67]	Retrospective study	China	December 2018 to October 2022	Advanced lung, gastrointestinal, urogenital, other cancers	904	Ethnicity not specified. Median age 65 years.	Anti-PD-1, anti-PD-L1	Not specified	Yes	No	9 of 16 (MINORS)

**Table 2:** Incidences of acute kidney injury (AKI) and nephritis in patients treated with ICIs in monotherapy and in combination. SEM, standard error of the mean.

Anti-PD-1				
**Study Identification**	Drug (Posology)	Patients Included	AKI Incidence (%)	Nephritis Incidence (%)
Abdelrahim et al., 2021 [19]	Nivolumab (not described)	331	3.93	
Antonia et al., 2016 [20]	Nivolumab (3 mg/kg every 2 weeks)	98	0.00	
Atkins et al., 2023 [23]	Nivolumab (240 mg every 2 weeks)	35	5.71	
Espi et al., 2021 [27]	Nivolumab (not described)	230	3.91	
Flippot et al., 2019 [28]	Nivolumab (3 mg/kg every 2 weeks)	73	4.11	
Grimm et al., 2022 [31]	Nivolumab (3 mg/kg every 2 weeks, 240 mg every 2 weeks, or 480 mg every 4 weeks)	228	1.32	
Julien et al., 2020 [36]	Nivolumab (0.1 mg/kg, 0.3 mg/kg, 1 mg/kg, 3 mg/kg, or 10 mg/kg every 2 weeks, then all patients 3 mg/kg every 2 weeks)	262	0.76	0.00
McFarlane et al., 2020 [40]	Nivolumab (240 mg every 2 weeks)	97		3.09
Meraz-Muñoz et al., 2020 [41]	Nivolumab (not described)	54	9.26	
Mourey et al., 2024 [43]	Nivolumab (3 mg/kg every 2 weeks)	720	1.11	
Rassy et al., 2022 [50]	Nivolumab (3 mg/kg every 2 weeks)	511	0.39	
Sharma et al., 2016 [55]	Nivolumab (3 mg/kg every 2 weeks)	78	1.28	
Spillane et al., 2020 [56]	Nivolumab (not described)	374	3.48	
Tio et al., 2018 [60]	Nivolumab (not described)	12	8.33	
Tomita et al., 2019 [61]	Nivolumab (3 mg/kg every 2 weeks)	410	3.90	
Verhaart et al., 2021 [64]	Nivolumab (3 mg/kg every 2 weeks)	264		2.27
Vogelzang et al., 2020 [65]	Nivolumab (240 mg every 2 weeks)	44		2.27
Zhao et al., 2021 [66]	Nivolumab (1.7 mg/kg or 2.7 mg/kg every 2 weeks)	32		3.13
**Nivolumab** **Incidence (weighted average ± SEM)**			**2.28 ± 0.03**	**1.57 ± 0.05**
Abdelrahim et al., 2021 [19]	Pembrolizumab (not described)	436	2.06	
Espi et al., 2021 [27]	Pembrolizumab (not described)	70	5.71	
Goldberg et al., 2020 [30]	Pembrolizumab (10 mg/kg every 2 weeks)	42	2.38	
Meraz-Muñoz et al., 2020 [41]	Pembrolizumab (not described)	36	58.33	
Mok et al., 2019 [42]	Pembrolizumab (200 mg every 3 weeks)	636	0.47	
Powles et al., 2022 [48]	Pembrolizumab (200 mg every 3 weeks)	488	4.10	0.20
Raghav et al., 2022 [49]	Pembrolizumab (200 mg every 3 weeks)	25	4.00	
Reck et al., 2016 [52]	Pembrolizumab (200 mg every 3 weeks)	154	1.95	
Spillane et al., 2020 [56]	Pembrolizumab (not described)	531	2.82	
Tio et al., 2018 [60]	Pembrolizumab (not described)	21		4.76
**Pembrolizumab** **Incidence (weighted average ± SEM)**			**3.18 ± 0.14**	**0.39 ± 0.04**
Cortazar et al., 2020 [11]	Indeterminate (nivolumab/pembrolizumab)	377	33.69	
Hofmann et al., 2016 [34]	Indeterminate (nivolumab 3 mg/kg every 2 weeks/pembrolizumab 2 mg/kg every 3 weeks)	496		0.40
Martini et al., 2018 [38]	Indeterminate anti-PD-1 (not described)	11		9.09
Noronha et al., 2021 [44]	Indeterminate (nivolumab 3 mg/kg every 2 weeks/240 mg flat dose every 2 weeks/pembrolizumab 200 mg every 3 weeks)	155		3.87
O’Reilly et al., 2020 [45]	Indeterminate (nivolumab/pembrolizumab)	17		5.88
Seethapathy et al., 2019 [54]	Indeterminate (nivolumab/pembrolizumab)	701	2.28	
Sukari et al., 2019 [58]	Indeterminate (nivolumab/pembrolizumab)	168	48.81	
Kanz et al., 2016 [37]	Indeterminate (nivolumab/pembrolizumab)	27		7.41
Zhou et al., 2024 [67]	Indeterminate anti-PD-1 (not described)	884	4.98	
**Anti-PD-1 total incidence (weighted average ± SEM)**			**5.32 ± 0.11**	**1.30 ± 0.04**
**Anti-PD-L1**				
Abdelrahim et al., 2021 [19]	Atezolizumab (not described)	22	0.00	
Sternberg et al., 2019 [57]	Atezolizumab (1200 mg every 3 weeks)	997	6.02	
Tio et al., 2018 [60]	Atezolizumab (not described)	2	0.00	0.00
**Atezolizumab** **Incidence (weighted average ± SEM)**			**5.88 ± 0.03**	
Apolo et al., 2020 [21]	Avelumab (10 mg/kg every 2 weeks)	249	10.04	
Powles et al., 2020 [47]	Durvalumab (1500 mg every 4 weeks)	345	0.58	
Massard et al., 2016 [39]	Durvalumab (10 mg/kg every 2 weeks)	61	1.64	
**Durvalumab** **Incidence (weighted average ± SEM)**			**0.74 ± 0.02**	
Martini et al., 2018 [38]	Indeterminate anti-PD-L1 (not described)	1		0.00
Seethapathy et al., 2019 [54]	Indeterminate (atezolizumab, avelumab, durvalumab)	37	2.70	
Zhou et al., 2024 [67]	Indeterminate anti-PD-L1 (not described)	20	10.00	
**Anti-PD-L1 total incidence (weighted average ± SEM)**			**5.25 ± 0.07**	**0.00 ± 0.00**
**Anti-CTLA-4**				
Abdelrahim et al., 2021 [19]	Ipilimumab (not described)	474	8.23	
Cortazar et al., 2020 [11]	Ipilimumab (not described)	92	47.83	
Meraz-Muñoz et al., 2020 [41]	Ipilimumab (not described)	219	11.42	
O’Reilly et al., 2020 [45]	Ipilimumab (not described)	59		3.39
Polkowska et al., 2019 [46]	Ipilimumab (not described)	333	5.71	
Seethapathy et al., 2019 [54]	Ipilimumab (not described)	249	4.82	
Spillane et al., 2020 [56]	Ipilimumab (not described)	590	2.54	
**Ipilimumab** **Incidence (weighted average ± SEM)**			**7.87 ± 0.21**	
Campbell et al., 2021 [25]	Tremelimumab (10 mg/kg every 4 weeks)	14	0.00	
Rini et al., 2011 [53]	Tremelimumab (6 mg/kg, 10 mg/kg, or 15 mg/kg every 12 weeks)	9	11.11	
**Tremelimumab** **Incidence (weighted average ± SEM)**			**4.35 ± 1.16**	
**Anti-CTLA-4 total incidence (weighted average ± SEM)**			**7.83 ± 0.21**	
**Anti-PD-1 + Anti-CTLA-4**				
Abdelrahim et al., 2021 [19]	Nivolumab + ipilimumab (not described)	159	11.32	
Antonia et al., 2016 [20]	Nivolumab (1 mg/kg) + ipilimumab (1 mg/kg), then nivolumab (1 mg/kg) + ipilimumab (3 mg/kg) or nivolumab (3 mg/kg) + ipilimumab (1 mg/kg) every 3 weeks	115	0.87	
Atkins et al., 2023 [23]	Nivolumab 3 mg/kg + ipilimumab 1 mg/kg, every 3 weeks	17	0.00	
Blas et al., 2024 [24]	Nivolumab + ipilimumab (not described)	2294	3.92	2.44
Dizman et al., 2022 [26]	Nivolumab 3 mg/kg + ipilimumab 1 mg/kg every 3 weeks	10	10.00	
George et al., 2022 [29]	Nivolumab 6 mg/kg + ipilimumab 1 mg/kg every 8 weeks	106	11.32	1.89
Gul et al., 2020 [32]	Nivolumab + ipilimumab (not described)	45		2.22
Hammers et al., 2017 [33]	Nivolumab (3 mg/kg) + ipilimumab (1mg/kg), nivolumab (1 mg/kg) + ipilimumab (3 mg/kg), or nivolumab (3 mg/kg) + ipilimumab (3 mg/kg) every 3 weeks	100	15.00	
Izumi et al., 2023 [35]	Nivolumab 240 mg + ipilimumab 1 mg/kg i.v. every 3 weeks	129	5.43	
Meraz-Muñoz et al., 2020 [41]	Nivolumab + ipilimumab (not described)	23	17.39	
Ready et al., 2023 [51]	Nivolumab 240 mg + ipilimumab 1 mg/kg every 6 weeks	391		0.77
Seethapathy et al., 2019 [54]	Nivolumab + ipilimumab (not described)	29	3.45	
Spillane et al., 2020 [56]	Nivolumab + ipilimumab (not described)	389	0.77	
Tachibana et al., 2021 [59]	Nivolumab + ipilimumab (not described)	30	6.66	
Tio et al., 2018 [60]	Nivolumab + ipilimumab (not described)	6	0.00	0.00
Tykodi et al., 2022 [62]	Nivolumab 3 mg/kg + ipilimumab 1 mg/kg every 3 weeks	52		3.85
Vasudev et al., 2024 [63]	Nivolumab 3 mg/kg + ipilimumab 1 mg/kg every 12 weeks	192	5.73	
**Nivolumab + ipilimumab** **Incidence (weighted average ± SEM)**			**4.58 ± 0.05**	**2.21 ± 0.01**
Abdelrahim et al., 2021 [19]	Pembrolizumab + ipilimumab (not described)	242	21.07	
Atkins et al., 2018 [22]	Pembrolizumab (2 mg/kg) + ipilimumab (1 mg/kg) every 3 weeks	22	0.00	
Tio et al., 2018 [60]	Pembrolizumab + ipilimumab (not described)	5	0.00	0.00
**Pembrolizumab + ipilimumab** **Incidence (weighted average ± SEM)**			**18.96 ± 0.39**	
**Anti-PD-1 + anti-CTLA-4 total incidence (weighted average ± SEM)**			**5.58 ± 0.08**	**2.21 ± 0.01**
**Anti-PD-L1 + Anti-CTLA-4**				
Powles et al., 2020 [47]	Durvalumab (1500 mg) + tremelimumab (75 mg) every 4 weeks	340	1.18	

## Data Availability

The raw data supporting the conclusions of this article will be made available by the authors on request.

## Abbreviation

AKI	Acute kidney injury
AKT	Serine-threonine kinase
CTLA-4	Cytotoxic T lymphocyte-associated protein 4
FDA	United States Food and Drug Administration
ICIs	Immune checkpoint inhibitors
IgG1	G1 immunoglobulin
IgG2	G2 immunoglobulin
IgG4	G4 immunoglobulin
PD-1	Programmed cell death protein 1
PD-L1	Ligand 1 of programmed cell death protein
PI3K	Inositolphosphatidyl-3-kinase
PP2A	Phosphatase protein 2A
